# Distinction of chia varieties in vivo and in vitro based on the flow cytometry and rosmarinic acid production

**DOI:** 10.1007/s00253-024-13171-w

**Published:** 2024-05-20

**Authors:** Sara Motyka, Agnieszka Szopa, Sergio J. Ochatt

**Affiliations:** 1 Chair and Department of Pharmaceutical Botany, Medical College, Jagiellonian University, Medyczna 9, 30-688, Kraków, Poland; 2 Doctoral School of Medical and Health Sciences, Medical College, Jagiellonian University, Łazarza 16, 31-530 Kraków, Poland; 3 INRAE, Institut Agro, Univ. Bourgogne, Univ. Bourgogne Franche-Comté, F-21000 AgroécologieDijon, France

**Keywords:** Chia, Flow cytometry, Genome size, Ploidy level, Secondary metabolites

## Abstract

**Abstract:**

Flow cytometry has made a significant contribution to the study of several complex fundamental mechanisms in plant cytogenetics, becoming a useful analytical tool to understand several mechanisms and processes underlying plant growth, development, and function. In this study, the genome size, DNA ploidy level, and A-T/G-C ratio were measured for the first time for two genotypes of chia, *Salvia hispanica*, an herbaceous plant commonly used in phytotherapy and nutrition. This study also evaluated, for the first time by flow cytometry, the capacity to produce organic acids of tissues stained with LysoTracker Deep Red after elicitation with either yeast extract or cadmium chloride. Rosmarinic acid content differed between the two chia varieties treated with different elicitor concentrations, compared with non-elicited plant material. Elicited tissues of both varieties contained a higher content of rosmarinic acid compared with non-elicited cultures, and cadmium chloride at 500 μM was much better than that at 1000 μM, which led to plant death. For both genotypes, a dose-response was observed with yeast extract, as the higher the concentration of elicitor used, the higher rosmarinic acid content, resulting also in better results and a higher content of rosmarinic acid compared with cadmium chloride. This study demonstrates that flow cytometry may be used as a taxonomy tool, to distinguish among very close genotypses of a given species and, for the first time in plants, that this approach can also be put to profit for a characterization of the cytoplasmic acid phase and the concomitant production of secondary metabolites of interest in vitro, with or without elicitation.

**Key points:**

*• Genome size, ploidy level, A-T/G-C ratio, and cytoplasm acid phase of S. hispanica*

*• Cytometry study of cytoplasm acid phase of LysoTracker Deep Red-stained plant cells*

*• Yeast extract or cadmium chloride elicited rosmarinic acid production of chia tissues*

**Graphical Abstract:**

**Figure:**
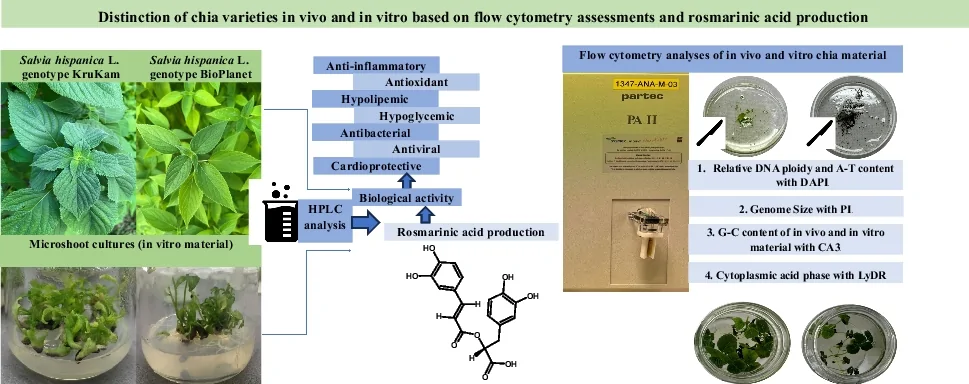

**Supplementary Information:**

The online version contains supplementary material available at 10.1007/s00253-024-13171-w.

## Introduction

In recent years, interest in medicinal plants increased significantly, due to their genetic diversity and the evolutionary relationships among species (Bai et al. 2013; Zhang et al. 2013). Thus, the mechanisms of genome size evolution and the relationship between genome size and geographic and ecological factors are presently the object of intense research, and there is a need for methods that will provide a thorough understanding of the genotypic and phenotypic traits of various species. Flow cytometry (FCM) is one such method, and its use is currently generating a large interest among plant researchers. FCM allows a fast and accurate study of genome size and ploidy levels, while simultaneously enabling to efficiently detect even small differences in DNA content. In this respect, although nuclear DNA content or genome size is an important character in the evaluation of biodiversity, these parameters have only been estimated for a rather small number of species to date (Ochatt 2008; Kelly and Leitch 2011; McKinnon 2018). The first applications of ploidy focused mainly on crop plants, and plant breeding dominates in this area. The three main areas of applied studies by FCM are the characterization of ploidy level in analyzed plant material; the control of ploidy stability at various stages during breeding programs, including in vitro culture; and the testing of desired cytotypes following their hybridization and/or ploidy manipulation (Sree Ramulu et al. 1986; Bartoš et al. 2005; Loureiro et al. 2008; Ochatt et al. 2011; Ochatt and Segui-Simarro 2021). In addition, recently, there has been a significant increase in the number of studies estimating the genome size and its impact on phenotypic and developmental traits, both at the subcellular, cellular, tissue, and organ levels (Grime 1998; Bennett 2000; Munzbergova 2009).

On the other hand, the study of several other traits of physiological and developmental importance, hitherto limited mainly to microscopic assessment and applied only to animal species (such as the triggering of apoptosis or endoplasmic reticulum stress due to an accumulation of acid phase organelles), is lately starting to be assessed also by FCM and extended to plants as well. In this context, there are several specific dyes to study cell compartments such as lysosomes, endosomes, or organelles, including mitochondria. These dyes are membrane-permeable weak bases which are linked to a fluorophore, and most probably have an affinity to acidic compartments because of protonation. LysoTracker is a group of such dyes that is available in different colors, among which LysoTracker Deep Red is used to stain acidic compartments such as lysosomes in viable animal cells (neurons and cancer cells among others) to study the localization and resident proteins of organelles, to assess their functionality, and to quantify their numbers. Unfortunately, records of its use in microscopic studies of plant cells in the literature are scanty, and there is none on its evaluation through FCM with plant cells to date.

Chia, *Salvia hispanica* L., is gaining popularity for its valuable medicinal properties. It is an herbaceous plant, belonging to the genus *Salvia* (sage) in the family *Lamiaceae* (Cahill 2003; Ayerza and Coates 2005; Motyka et al. 2021, 2022, 2023a, b), which includes about 900 species that occur almost all over the world, mainly in North, Central, and South America; in South Africa and Southeast Asia; and also in Europe (Lu and Yeap Foo 2002; Cahill 2003; Das 2017). The current 10th edition of European Pharmacopoeia (2020) presents monographs of raw materials obtained from various species of *Salvia* spp., namely, *S. lavandulifolia* (lavender sage), *S. miltiorrhiza* (red root sage or red sage), *S. officinalis* (medical sage), *S. sclarea* (clary sage), and *S. triloba* (Greek sage). All monographs confirm that *Salvia* spp. are valuable medicinal species, with health-promoting properties used in therapeutic treatments (Perry et al. 2010; Motyka et al. 2023a, b). In *S. hispanica*, the main raw materials obtained are chia seeds (*Salviae hispanicae semen*), which have been used for ages in Traditional Chinese Medicine (TCM) and in traditional Latin American Medicine (Cahill 2003; Melo et al. 2019; Sánchez-Velázquez et al. 2022) Nowadays, other parts of *S. hispanica* plants, mainly the sprouts, leaves, flowers, and herb, are also becoming increasingly popular in many sectors of the food, pharmaceutical, and therapeutic industries (Kojima 1996; Hasler 1998; Arai 2002; Motyka et al. 2022, 2023a, b). Nonetheless, chia seeds remain the most well-known product from *S. hispanica*, which has been approved by the European Food Safety Authority (EFSA) and whose market position is well-established, since they are an important ingredient in food production (Bresson et al. 2009; EFSA NDA Panel 2019). Interestingly, though, it is noteworthy that *S. hispanica* is classified as a neglected plant in highly industrialized countries, while it is considered a valuable product for “medium- and high-income countries” (Ochatt and Jain 2007; Alkhatib et al. 2017; Ali and Rahut 2019). Nowadays, chia seeds are gaining popularity and are valued for their nutritional qualities. They are a source of protein, dietary fiber, lipids, vitamins, and minerals. The special health properties of chia seeds are determined by the presence of omega-3 fatty acids. Moreover, chia seeds owe their bioactive potential to the presence of antioxidant phenolic compounds (Oteri et al. 2022; Agarwal et al. 2023; Ferreira et al. 2023). Polyphenols, mainly cinnamic acid derivatives and flavonoids, are responsible for the antioxidant activity of chia seeds (Knez Hrnčič et al. 2019; Aja and Haros 2022; Ghafoor et al. 2022; Agarwal et al. 2023).

Various *Salvia* species have been analyzed using FCM in the past but there are no such studies on *S. hispanica* L. to date. This work represents for the first time the comparison and examination of the species *S. hispanica* from a genetic, phylogenetic, and qualitative point of view, compared with other more commonly studied species of the genus *Salvia*. In this study, the genome size, DNA ploidy level, and A-T/G-C ratio were measured for the first time for two genotypes of chia, *S. hispanica*, an herbaceous plant commonly used in phytotherapy. This study aimed to determine the size of the *S. hispanica* genome in comparison with other species of the genus *Salvia*. Moreover, for the first time in plants, this study evaluated by flow cytometry the capacity of elicited cells to produce rosmarinic acid compared with control plants, through the analysis of cells stained with LysoTracker Deep Red. The study demonstrates that in vitro plant cultures of *S. hispanica* can be an innovative and useful source of secondary metabolites compared to plant material grown in vivo.

## Material and methods

### Plant material

The seeds of the plant material analyzed were obtained from KruKam Polska S.A. (21B Marklowicka St., 44-300 Wodzisław Śląski, Poland), and grown in Guatemala, and from Bio Planet Polska S.A. (Wilkowa Wieś 7, 05-084 Leszno, Poland), which were organically grown in Paraguay.

The *S. hispanica* plants were cultivated under greenhouse conditions in the Prof. Marian Koczwara Medicinal Plants Garden of the Faculty of Pharmacy at the Jagiellonian University Medical College in Cracow, Poland (geographical location—latitude: 50 01′ 12″ N, longitude: 19 99′ 45″ E). The leaves were harvested in August 2021, during the flowering and fruiting of the plants. For the analyses, this study used *S. hispanica* leaves obtained from in vivo grown plants and plant material from in vitro conditions. The phenotype of in vivo and in vitro material of the two chia genotypes studied in this work, henceforth called KruKam and Bio Planet, is illustrated in Supplementary Figure S1.

### Experimental in vitro cultures

All procedures were carried out under aseptic conditions (laminar flow sterile air box, type KL-21, POLON, Poznań, Poland), using autoclaved utensils and vessels (type ASVE Spółdzielnia Pracy Mechaników, Warsaw, Poland). For surface sterilization, seeds were immersed in a 0.2% mercury (II) chloride (HgCl_2_) solution for 5 min and rinsed five times with sterile distilled water. The disinfected seeds of two commercial *S. hispanica* varieties were transferred to Murashige and Skoog (1962; MS) medium supplemented with 2 mg/L BP (6-benzyladenine), 30 g/L sucrose, and 7 g/L agar for initiation, and 1 g of fresh weight (FW) of *S. hispanica* shoots was subcultured monthly thereafter. All cultures were grown under continuous light-emitting diode (LED) white light (2.75 W/m^2^, photosynthetic photon flux density (PPFD) of 40 µmol m^−2^ s^−1^) at a temperature of 25 ± 2 °C.

### Elicitation procedure

Stock solutions of CD (CdCl_2_, cadmium chloride, at concentrations of 500 µM or 1000 µM) and YeE (yeast extract at concentrations of 500 mg/L or 1000 mg/L) were prepared in 250 mL distilled H_2_O and were filter-sterilized through a 0.22-µm syringe filter (Millex^®^GP; Merck Millipore, Burlington, MA, USA).

Elicitors from the stock solutions were added to cultures by day 21 of growth, at 500 and 1000 µM CdCl_2_ (Sigma-Aldrich, St. Louis, MO, USA) for CD treatment and 500 and 1000 mg/L yeast extract (YeE) (Sigma-Aldrich, St. Louis, MO, USA) for YeE treatments. The media and biomass samples were collected 7 days after elicitation.

Microshoots grown in the absence of elicitors were used as control (C), and on day 10 of growth, 5 mL (as for the elicitors) of double distilled sterile water was added. The control samples were collected at the same time points as those of the experimental cultures.

### Chromatographic analyses of rosmarinic acid

The rosmarinic acid content was assessed in extracts prepared from 100 mg of DW (dry weight) tissue in 2 mL of HPLC-grade methanol. Tissue was extracted by sonication (2 × 30 min at 23 ± 2 °C) (ultrasonic bath, Polsonic, Warsaw, Poland). Extracts were centrifuged (25,255×g for 8 min, 4 °C), with samples filtered through Millex^®^GP 0.22-μm syringe filters (Millipore, Merck, Darmstadt, Germany) for analysis by diode array detector high-pressure liquid chromatography (DAD-HPLC). The validated method was applied for quantitative analyses of rosmarinic acid using Merck-Hitachi (LaChrom Elite, Tokyo, Japan) apparatus and on a RP-18 column (Purospher, 5 μm, 250 × 4 mm; Merck, Darmstadt, Germany) (Sułkowska-Ziaja et al. 2016). The parameters were as follows: flow rate 1 mL/min, A—methanol, 0.5% acetic acid 1:4, B—methanol (v/v), temp. 25 °C, injection 10 µL, *λ* = 254 nm. The gradient program was as follows: 0 to 20 min, 0% B; 20 to 35 min, 0–20% B; 35 to 45 min, 20–30% B; 45 to 55 min, 30–40% B; 55 to 60 min, 40–50% B; 60 to 65 min, 50–75% B; and 65 to 70 min, 75–100% B. Identification was done by comparison to Rt (retention time) and UV spectra of reference substance of rosmarinic acid (Sigma-Aldrich Co, Merck, Darmstadt, Germany). Assays were done in three replications, with results expressed in mg/100 g DW ± SD.

### Flow cytometry analyses

For flow cytometry assessments, a single leaf from a seedling from in vitro or in vivo cultures of *S. hispanica* was taken and chopped in nuclei extraction buffer as previously reported (Galbraith et al. 1983; Ochatt et al. 2013). All analyses were performed using a Partec PAS-II flow cytometer (Sysmex Europe GmbH, Norderstedt, Germany) equipped with an argon laser source and an HBO-100 W mercury lamp with a dichroic mirror (TK420) and with plant material excited as appropriate for the stain tested. For the assessment of DNA content and ploidy level, leaves from regenerated shoots in vitro and from plants grown in vivo of chia were run simultaneously with leaves from pea (*P. sativum*) cv. Cameor as the internal standard.

#### Assessments of A-T content

The A-T content of DNA was assessed with nuclei isolated mechanically by chopping the tissues in 1 mL of DNA 1 Step Nucleus Extraction Buffer (Sysmex Europe GmbH, Norderstedt, Germany), then diluted with a further 1 mL of buffer to provide 2 µg DAPI (4′,6-diamidinedihydrochloran-2-phenylindone)/mL of nuclei suspension and sieved through a 50-µm Celltrix nylon mesh (Sysmex Europe GmbH, Norderstedt, Germany) prior to the readings. Being a non-intercalating dye, once excited under the UV, DAPI attaches to DNA in the minor groove of the A-T-rich sequences (Kapuscinski 1995) and forms a blue fluorescing complex which permits to calculate the A-T content using the formula reported by Marie and Brown (1993), while the cell cycle was analyzed automatically using the Flomax program (Partec GmbH, Jettingen-Scheppach, Germany), and the mitotic index and average *C* value were calculated as previously reported (Ochatt 2008; Ochatt et al. 2013; Eicher et al*.* 2022).

#### Assessments of genome size

To assess the genome size, the DNA intercalating dye propidium iodide (PI) was excited with a laser line of 561 nm and with maximum emission of 636 nm as described (Ochatt et al. 2013; Eicher et al. 2022), with tissues chopped in 400 µL of CyStain PI Absolute P nuclei extraction buffer (Sysmex Europe GmbH, Norderstedt, Germany) to which 1.6 mL of staining buffer containing PI was added. PI is assumed not to be base pair-specific but was reported to exhibit GC preference by Vinogradov (1994). Nuclei extracted from leaves of in vitro grown plants of *P. sativum* L. (pea) cv. Cameor, of a known genome size of 4.90 pg DNA/1 C, were run simultaneously as the internal standard. The chia genome size was calculated according to Doležel et al. (2003), where 1 pg DNA = 978 Mpb.

#### Assessments of G-C content

The determination of the G-C content of samples (Meister and Barow 2007) was performed as reported by Eicher et al. (2022), with leaves chopped in 500 µL of fresh McIlvaine (1921) buffer at pH 7.0, with chromomycin A3 (CA3, at 0.5 µg/mL of nuclei suspension) dissolved in the same buffer containing 0.02 M MgCl_2_, and with samples kept on cold ice or in the fridge for 15 min prior to readings under laser excitation. The Mg^2+^ ions added to nuclei suspended in the buffer after dye adjunction allow the G-C specific CA3 dye to form complexes with helical DNA (Eicher et al*.* 2022). The A-T/G-C ratio was calculated with the formula reported by Godelle et al*.* (1993) and modified by Marie and Brown (1993). The A-T% and G-C% were determined separately and were then recalculated to add up to 100%.

#### Flow cytometry determination of the cytoplasm acid phase in control and elicited tissues of chia

A separate series of experiments was designed to evaluate the extent to which the elicitation of in vitro chia cultures with CD or YeE may increase the production by cells of acids of medicinal interest, including rosmarinic acid. In these experiments, for the first time in plants, cells were analyzed by flow cytometry after being stained with LysoTracker^®^ Deep Red (LyDR; Invitrogen Ref. L12492; Molecular Probes™, Waltham, MA, USA). This probe has an excitation wavelength maximum of 647 nm and emission maximum of 668 nm, and no washes are required before the readings/observations. Thus, 1 mM LyDR diluted in dimethyl sulfoxide (DMSO) (kept frozen) was pre-warmed to room temperature. Meanwhile, tissues were chopped in 1 mL of PBS at pH 7.4; then, PBS was gently pipetted away; and chopped tissues were stained with LyDR for 15 min at 37 °C or for 30 min at room temperature. LyDR stain was then diluted with 2 mL PBS, and stained tissues were sieved through a 50-µm nylon mesh Celltrix (Sysmex Europe GmbH, Norderstedt, Germany) prior to the readings. Assessments with LyDR were performed with a 640-nm laser line for excitation (using a Cy5 filter as for microscopic observations), and emission was recorded at 647/668 nm and 638-nm laser excitation (gain = 450, lower limit (LL) = 75, upper limit (UL) = 275, speed = 0.5 µL/s).

### Statistical analysis

All flow cytometry results were expressed as histograms where the number of particles counted (in ordinates) was plotted against the intensity of the epifluorescence they emitted in abscissa, using a linear scale for DAPI- and PI-stained nuclei, and on a logarithmic scale for cells stained with CA3 and LyDR. For samples stained with DAPI, PI, and CA3, assessments were repeated at least twice at 2-day intervals. Counting of stained nuclei included a minimum of 2500 nuclei per run (i.e., ≥ 7500 per sample) for DAPI, 5000 nuclei per run (i.e., ≥ 15,000 per sample) for PI, and 30,000 nuclei per run (i.e., 90,000 per sample) for CA3. For LyDR, analyses were repeated once for each sample analyzed and included about 6000 cells per reading (i.e., 12,000 per sample).

For all stains, when the raw profiles had their peaks analyzed, the in-built FloMax program (Sysmex Europe GmbH, Norderstedt, Germany) converted the means into medians, provided the % surface area underneath the resulting Gauss bells, gave the coefficient of variation (CV%) of both the peak position and surface area, and calculated the* Χ*^2^ for the analyzed samples. In addition, data were further analyzed statistically using the non-parametric Kruskal-Wallis test (followed by Dunn’s test) to determine the differences (*p* = 0.05) between samples.

Data of chromatographic estimations are expressed as means and standard deviation (SD). The collected data were subjected to a one-way analysis of variance (ANOVA) followed by Duncan’s post hoc test. A two-sided *p*-value of 0.05 was applied to declare statistical significance. Statistical analysis was performed by using the Statistica software program (StatSoft, Poland).

## Results

**Fig. 1:**
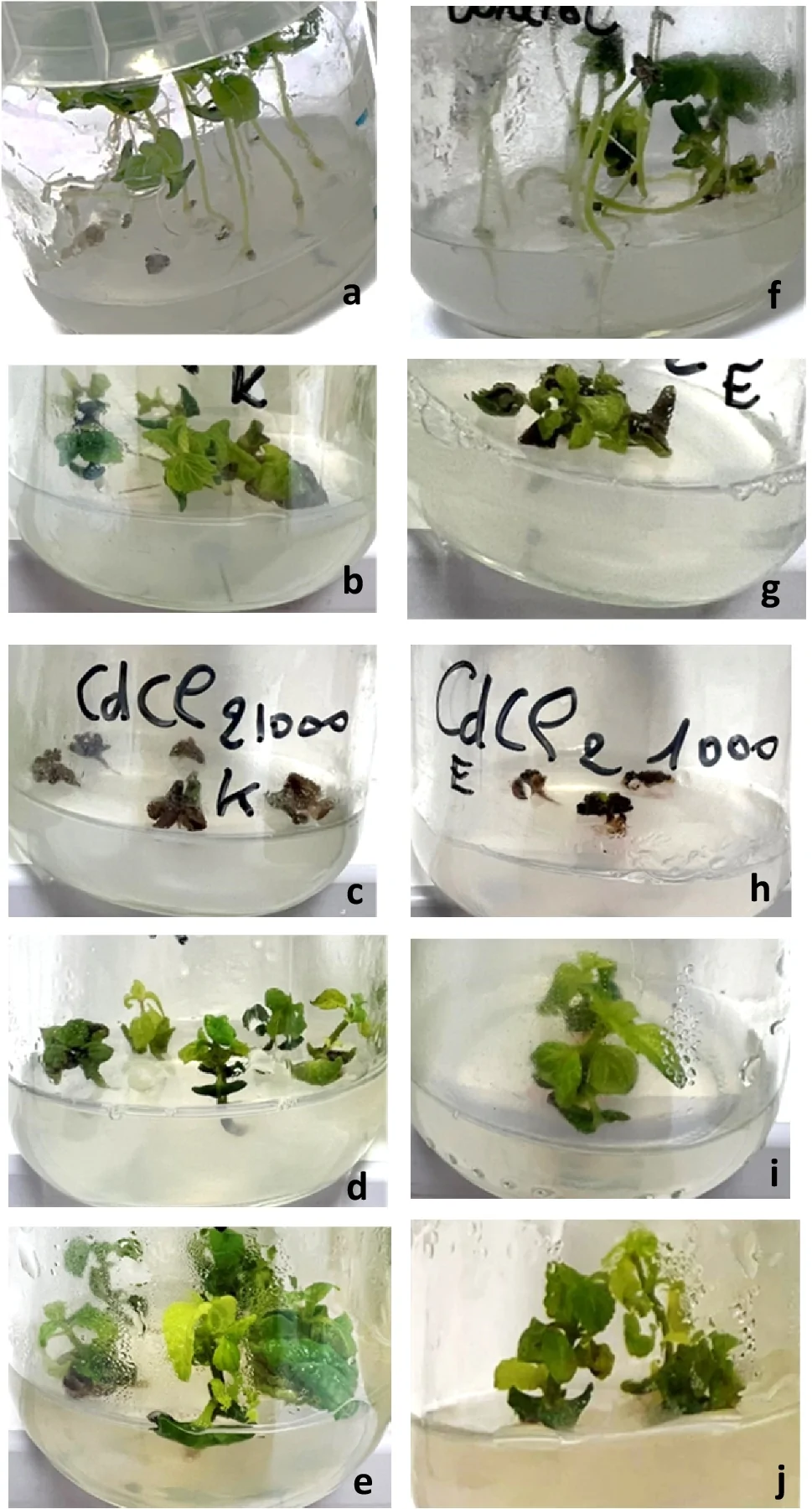
Representative microshoots of *S. hispanica* from genotypes KruKam (left, **a**–**e**) and Bio Planet (right, **f**–**j**) elicited with CD and YeE. KruKam: **a** non-elicited control, **b** 500 µM CD, **c** 1000 µM CD, **d** 500 mg/L YeE, **e** 1000 mg/L YeE. Bio Planet: **f** non-elicited control, **g** 500 µM CD, **h** 1000 µM CD, **i** 500 mg/L YeE, **j** 1000 mg/L YeE

A variation in the appearance of *S. hispanica* microshoot cultures was observed depending on the elicitor and concentration used compared with non-elicited cultures (Fig. 1 and Supplementary Fig. S2). In vitro cultures of *S. hispanica* obtained from KruKam had larger and greener leaves compared with genotype Bio Planet. KruKam grew more densely and had more branches, while Bio Planet genotype had more, thin, and taller stems. The application of 500 μM CD resulted in the maintenance of a similar appearance of in vitro cultures of both tested *S. hispanica* genotypes. On the other hand, a higher CD concentration (1000 μM) negatively affected the further growth and rosmarinic acid production in both varieties in vitro (Table 2). CD at 1000 μM was too strong and caused gradual decay/death and growth stoppage of the explants. YeE enhanced rosmarinic acid production at both concentrations used compared with the control. Moreover, after YeE elicitation with both 500 and 1000 mg/L, in vitro cultures of *S. hispanica* from both genotypes developed a more robust root system (Supplemental Fig. S2). Such phenotypic differences between these two closely related genotypes prompted the subsequent series of flow cytometry analyses on their ploidy level and genome size.

**Fig. 2:**
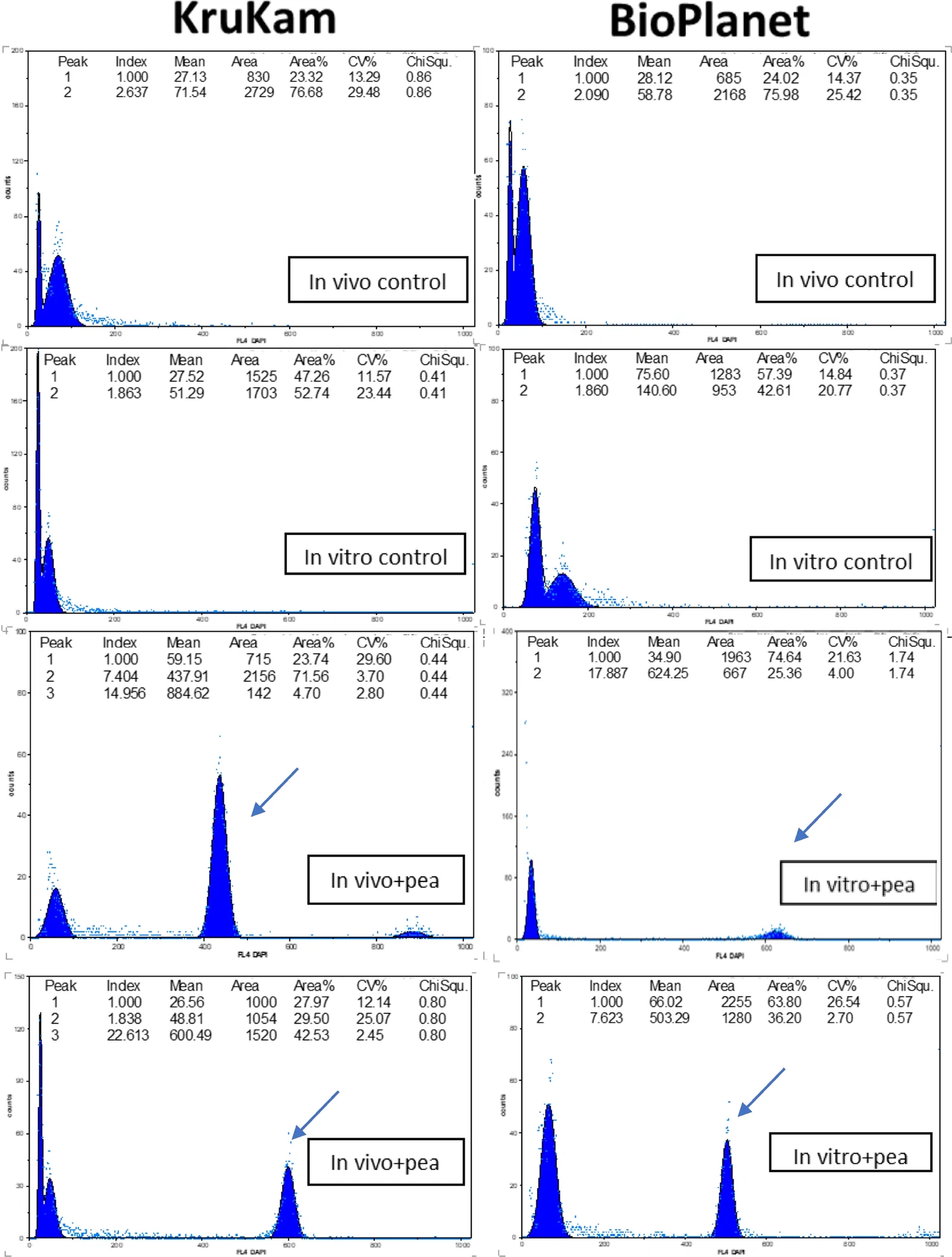
Flow cytometry assessments of relative DNA ploidy with DAPI. The peak for pea is arrowed

A normal, euploid profile includes two peaks, the first of which corresponds to the nuclei in G1 (2C DNA) and the second one with twice the channel value to those in G2/M (4C DNA). In these experiments, DAPI-stained nuclei of both chia genotypes were read alone and together with a nuclear suspension of pea as internal standard, as shown in Fig. 2. The results with DAPI staining indicated that Bio Planet would be a tetraploid, while KruKam would be diploid, and phenotypical differences observed between genotypes also validate this (Fig. 1 and Supplemental Fig. S2).

Interestingly, despite the difference observed in relative DNA ploidy, no such significant differences were apparent in terms of nuclear DNA content measured with PI. Moreover, both genotypes were very close in terms of genome size, with 0.4 pg 1C DNA for KruKam and 0.36 pg 1C DNA for Bio Planet. They also were the two genotypes with the smallest genome size of all other *Salvia* species reported to date, where 1C DNA content reported had a mean of 0.84 pg (with a minimum of 0.43 pg, a maximum of 2.05 pg, and a SD of 0.42 pg), as detailed in Table 1.

**Table 1:** The nuclear DNA content of *S. hispanica* compared with other species of the genus *Salvia. nd*, no data

Species	Locality	2 *C* value	Internal standard	Ploidy level	1 Cx value (pg)	1 *C* value	References
*S. hispanica* L. genotype KruKam	Guatemala (Mazatenango)	nd	*P. sativum* L. cv. Cameor (1C = 4.42 pg DNA)	2	0.40	nd	This work
*S. hispanica* L. genotype Bio Planet	Paraguay (Pilar)	nd	*P. sativum* L. cv. Cameor (1C = 4.42 pg DNA)	2	0.36	nd	This work
*S. pratensis* L.	nd	0.91	*Glycine* max Merr. ‘Polanka’ (2C = 2.5 pg)	2	0.46	nd	Münzbergová (2009)
*S. verticillata* L.	nd	1.37	*Glycine* max Merr. ‘Polanka’ (2C = 2.5 pg)	2	0.68	nd	Münzbergová (2009)
*S. brachyodon* Vandas	Croatia (Peljesac Peninsula)	0.95	*P. hybrida* (Hook) Vilm. cv. ‘PxPC6’	2	0.48	0.48 (464.55)	Maksimović et al. (2007)
*S. militiorhizza* L.	Slovenia (Stajerska)	4.10	*P. sativum* L. (1C = 4.42 pg DNA)	nd	nd	2.05	Zhang et al. (2013)
*S. glutinosa* L.	Bosnia Herzegovina (Sarajewo)	2.11	*P. hybrida* (Hook) Vilm. cv. ‘PxPC6’	2	0.53	1.06 (1031.79)	Siljak-Yakovlev et al. (2010)
*S. glutinosa* L.	nd	2.31	*P. hybrida* (Hook) Vilm. cv. ‘PxPC6’	2	1.18	1.055	Siljak-Yakovlev et al. (2010)
*S. nemerosa* L.	Bosnia Herzegovina (Sarajewo)	1.09	*P. hybrida* (Hook) Vilm. cv. ‘PxPC6’	2	0.55	0.55 (533.01)	Siljak-Yakovlev et al. (2010)
*S. officinalis* L.	Croatia (Peljesac Peninsula)	0.97	*P. hybrida* (Hook) Vilm. cv. ‘PxPC6’	2	0.49	0.49 (474.33)	Maksimović et al. (2007)
*S. ringens* var. *baldacciana* Briq. det. J. Bornmüller	Macedonia (Raec canyon)	1.22	*L. esculentum* Mill. cv. ‘Roma’	nd	nd	0.61 (596.58)	Siljak-Yakovlev et al. (2010)
*S. sclarea* L.	Croatia (Mt. Biokovo)	1.16	*P. hybrida* (Hook) Vilm. cv. ‘PxPC6’	nd	nd	0.58 (567.24)	Siljak-Yakovlev et al. (2010)
*S. verticillata* L.	Bosnia Herzegovina (Sarajewo)	1.40	*L. esculentum* Mill. cv. ‘Roma’	nd	nd	0.70 (684.60)	Siljak-Yakovlev et al. (2010)
*S. bracteata* Banks & Sol.	Lebanon (Majdel Tarchich)	nd	*P. hybrida* (Hook) Vilm. cv. ‘PxPC6’ (2.85 pq)	nd	nd	0.72	Bou Dagher-Kharrat et al. (2013)
*S. fruticosa* var. *libanotica* Mill.	Lebanon (Nahr Ibrahim)	nd	*P. hybrida* (Hook) Vilm. cv. ‘PxPC6’ (2.85 pq)	nd	nd	0.84	Bou Dagher-Kharrat et al. (2013)
*S. microstegia* Boiss. & Bal.	Lebanon (Faraya)	nd	*S. lycopersicum* L. “Montfavet 63-5” (1.99 pg)	nd	nd	0.63	Bou Dagher-Kharrat et al. (2013)
*S. multicaulis* Vahl var. *simplicifolia* Boiss.	Lebanon (Naheleh)	nd	*P. hybrida* (Hook) Vilm. cv. ‘PxPC6’ (2.85 pq)	nd	nd	1.13	Bou Dagher-Kharrat et al. (2013)
*S. viridis* L.	Lebanon (Ehden)	nd	*S. lycopersicum* L. “Montfavet 63-5” (1.99 pg)	nd	nd	0.43	Bou Dagher-Kharrat et al. (2013)
*S. viscosa* Jacq.	Lebanon (Ammatour)	nd	*P. hybrida* (Hook) Vilm. cv. ‘PxPC6’ (2.85 pq)	nd	nd	1.13	Bou Dagher-Kharrat et al. (2013)

**Fig. 3:**
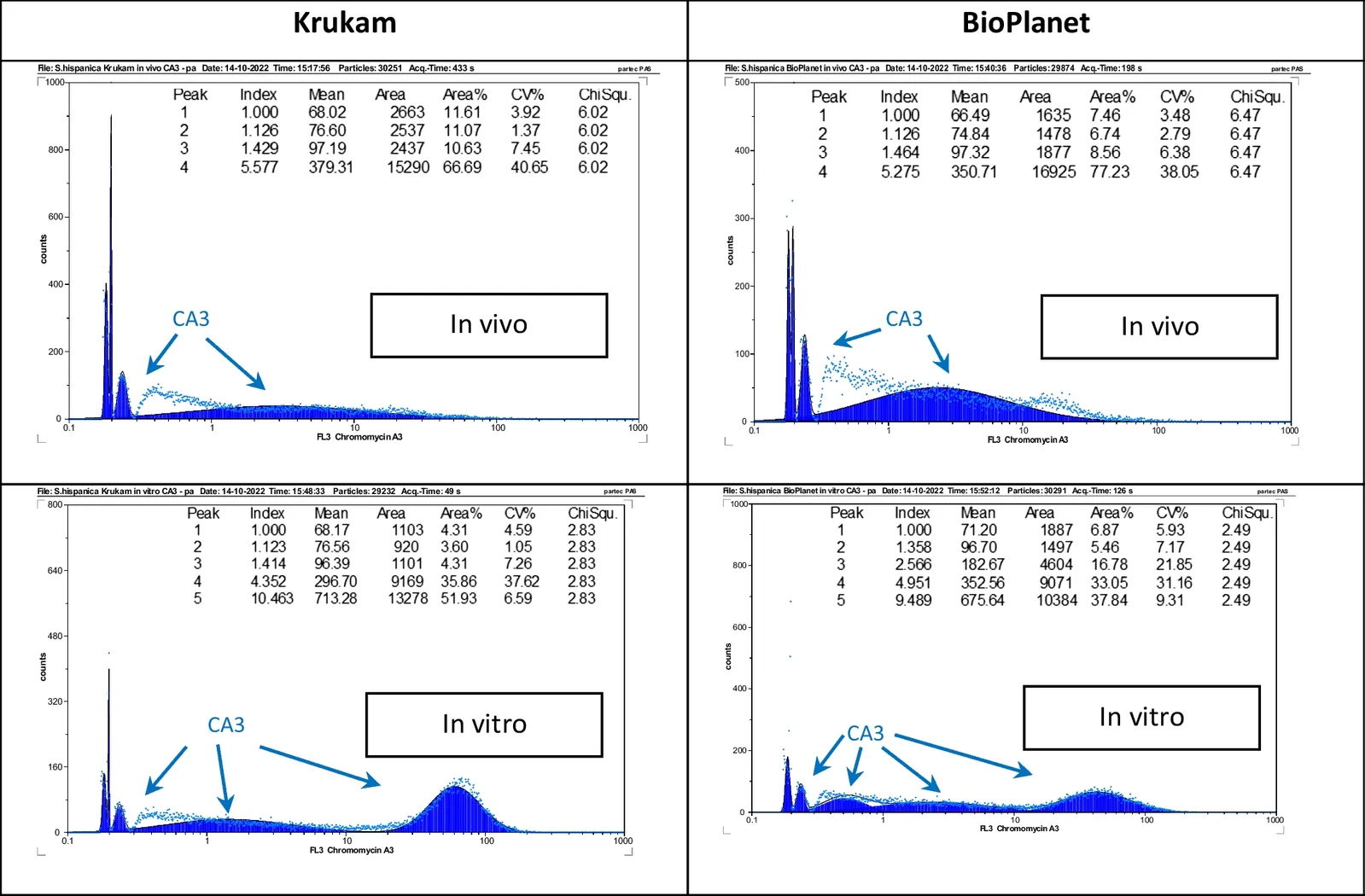
Comparison of in vivo and in vitro material of the two genotypes analyzed with CA3

**Fig. 4:**
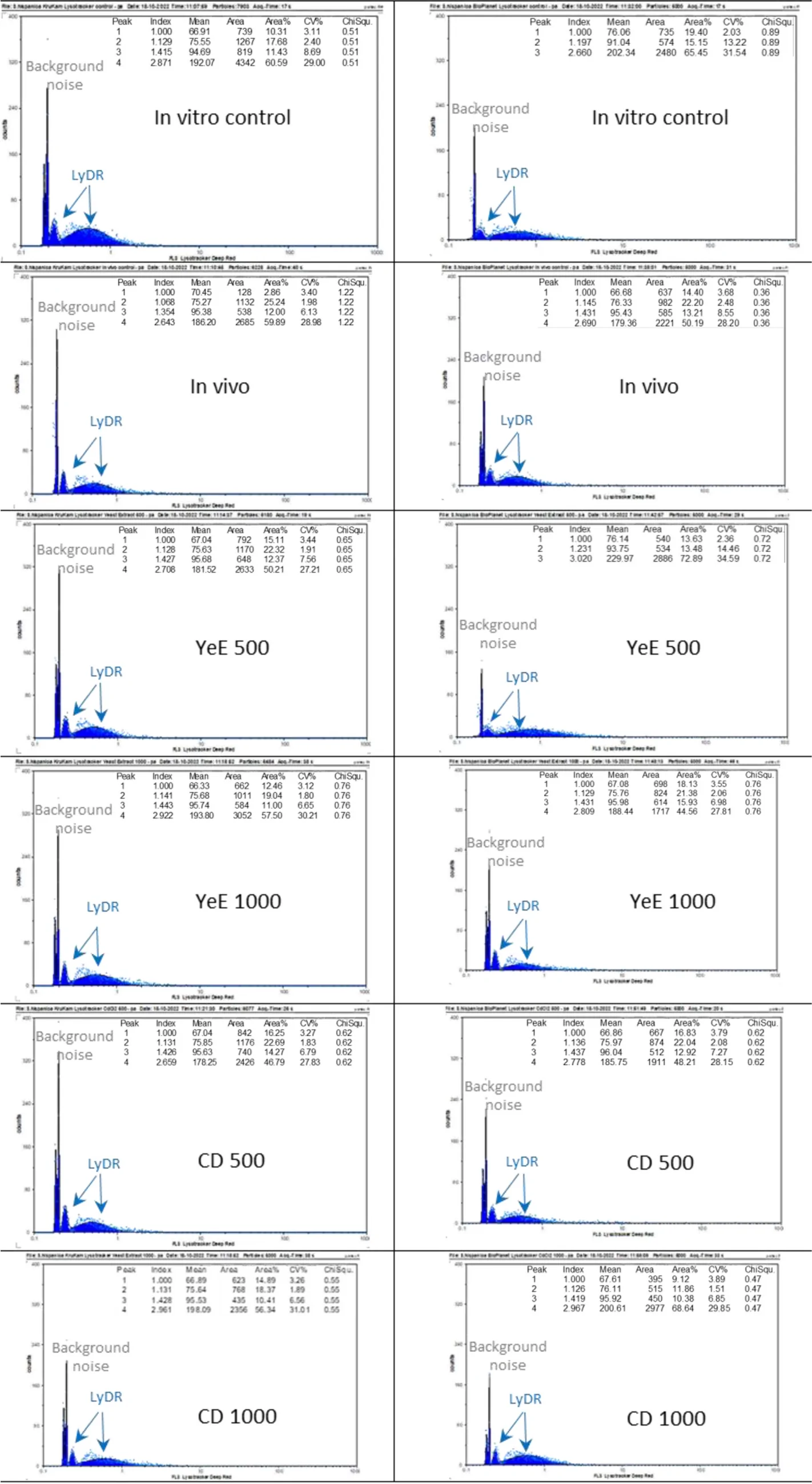
FCM profiles of tissues of KruKam and Bio Planet analyzed with LyDR

Measuring the G-C content is another means of distinguishing closely related genotypes, and the experiments undertaken with CA3 showed that genotype Bio Planet had much more G-C than KruKam (Fig. 3), which could be correlated with a larger capacity to produce secondary metabolites of interest. Likewise, tissues developed in vitro grew faster and exhibited a larger G-C content than the in vivo grown plants. In addition, from the results obtained with the different dyes used and applying the formulae described, the A-T% and G-C%, then the A-T/G-C ratio for the genotypes of *S. hispanica* studied was calculated, as detailed in Table 2. The G-C% values found when the pea cv. Cameor was used as reference were always slightly higher than those obtained for other *Salvia* species (Siljak-Yakovlev et al. 2010), but results were consistently reproducible with both genotypes analyzed, strengthening the usefulness of this approach for the taxonomical distinction between closely related genotypes. Eliciting tissue and cell cultures of medicinal plants in vitro is an efficient strategy to improve their ability to produce secondary metabolites, which is subsequently assessed by various physico-chemical methods as a standard practice. In this work, for the first time in plants, the acid phase of the cytoplasm of such cultured tissues was analyzed by flow cytometry using the stain LysoTracker Deep Red (LyDR) as an indirect measurement of their capacity to produce organic acids of medicinal interest, such as rosmarinic acid. LyDR is a cell-permeable, non-fixable, red fluorescent dye that stains lysosomes and other acidic compartments within cells such as autolysosomes. Avrahami et al. (2013) used LyDR to demonstrate relatively large elevations in pH as LyDR is a single wavelength dye, whereby any change observed in the amount of emitted light will result either from an increase in pH or from a change in the number and/or area of the lysosomes. In this respect, the interest in using the LyDR probe resides in its high selectivity for acidic organelles compared with other classical dyes such as neutral red or acridine orange (Chazotte 2011). The results obtained with the flow cytometry analyses of LyDR-stained tissues of chia of both genotypes revealed that, naturally, their cytoplasm acid phase was higher in vitro than in vivo, while Bio Planet produced more organic acids than KruKam (Fig. 4). It was also apparent that there were differences in terms of elicitation of acid phase between YeE and CD; YE1000 appeared to improve the acid phase in terms of median and surface area of the peaks obtained as compared with the in vivo and in vitro controls and also as compared with CD. Moreover, CD generally proved to be deleterious, since their viability was decreased at both 500 mM and 1000 mM as determined microscopically with FDA staining (data not shown). However, CD500 resulted in a 25% increase of the acid phase compared with the in vitro control.

**Table 2:** Comparison of G-C% between species of the genus *Salvia*

Taxa	Country of origin of seeds	GC%	References
*S. brachyodon*	Croatia (Peljesac Peninsula)	38.52	Siljak-Yakovlev et al. (2010)
*S. officinalis*	Croatia (Peljesac Peninsula)	38.55	Siljak-Yakovlev et al. (2010)
*S. hispanica* in vivo genotype KruKam	Paraguay	38.89	This work
*S. hispanica* in vivo genotype Bio Planet	Guatemala	38.83	This work
*S. hispanica* in vitro genotype KruKam	Paraguay	39.35	This work
*S. hispanica* in vitro genotype Bio Planet	Guatemala	38.79	This work

In vitro cultures obtained from the two genotypes were shown to contain a higher rosmarinic acid content than the tissues grown in vivo. The results also revealed that the rosmarinic acid content differs between the two *S. hispanica* varieties treated with different elicitors at different concentrations (Table 3). For both genotypes, CD at a concentration of 500 μM was better than that of 1000 μM in terms of rosmarinic acid production. The use of YeE as an elicitor resulted in better results and a higher content of rosmarinic acid compared with CD. The use of either elicitor at a lower concentration (500 μM CD, 500 mg/L YeE) resulted in a significant increase in rosmarinic acid content compared with the higher concentration used (1000 μM CD, 1000 mg/L YeE) as well as to the control. The content of rosmarinic acid was highest (4651.5 mg/100 g DW) for genotype KruKam elicited with CD at 500 μM and was more than 5 times higher than in the control (881.09 g/100 g DW). For genotype Bio Planet elicited with 500 µM CD, the content of rosmarinic acid was 3020.82 mg/100 g DW, i.e., almost 4 times higher compared with the non-elicited control (838.14 g/100 g DW). An inverse dependence was observed for tissues elicited with CD as a higher CD concentration significantly decreased rosmarinic acid content in both *S. hispanica* genotypes. Thus, in KruKam elicited with 1000 μM CD, the content of rosmarinic acid was 868.41 mg/100 g DW, i.e., almost 6 times lower compared with 500 μM CD and roughly the same rosmarinic acid content as found in the non-elicited microshoots. Conversely, for YeE, a higher concentration of elicitor resulted in a higher rosmarinic acid content in both *S. hispanica* genotypes. Upon elicitation with YeE at 1000 mg/L, the rosmarinic acid content was 3135.47 mg/100 g DW for KruKam and 4081.19 mg/100 g DW for Bio Planet, which was 2.5–5 times higher than that for the non-elicited cultures. Application of YeE at a lower concentration (500 mg/L) also increased rosmarinic acid content in both genotypes compared with the control, but to a lesser extent. Thus, for genotype KruKam, the content of rosmarinic acid was 1696.00 mg/100 g DW, twice that of the control, while for genotype Bio Planet elicited with YeE at concentration of 500 mg/L, the rosmarinic acid content was 2929.18 mg/100 g DW, hence 3.5 times that of non-elicited cultures. Table 3 shows that, in terms of the content of rosmarinic acid, elicitation with CD at 500 μM was better than that with 1000 μM for both genotypes of *S. hispanica*. The reverse was true for YeE, as the higher the concentration of elicitor used, the higher the rosmarinic acid content of tissues.

**Table 3:** Content (mg/100g DW ± SD) of rosmarinic acid in elicited genotypes of *S. hispanica*

Genotypes	Controls		Elicitors applied in in vitro			
	Control in vivo	Control in vitro	CD		YeE	
	Non-elicited	Non-elicited	500 (μM)	1000 (μM)	500 (mg/L)	1000 (mg/L)
KruKam	198.53 ± 0.18^a^	881.09 ± 1.02^b^	4651.46 ± 31.27^c^	868.41 ± 7.81^b^	1696.00 ± 16.29^d^	3135.47 ± 3.34^e^
Bio Planet	175.28 ±0.57^a^	838.14 ± 8.23^b^	3020.82 ± 7.60^e^	536.79 ± 11.51^f^	2929.18 ± 9.58^e^	4081.19 ± 24.72^c^

Different letters as superscripts denote significant differences at *p* = 0.05

## Discussion

In this study, the genome size, DNA ploidy level, and A-T/G-C ratio were measured for the first time for two genotypes of chia, *S. hispanica*. Moreover, for the first time in plants, the capacity to produce organic (including rosmarinic) acids of cells elicited with either YeE or CD was also evaluated by FCM using LyDR stain.

FCM has become a major approach with analytical capability for understanding the mechanisms and processes underlying plant growth, development, and function. As such, it has had a large impact on many fields of knowledge, from cell biology to genetics, immunology, molecular biology, and environmental science (Ochatt 2008). The ability to isolate a large number of particles of the same type provides sufficient material for subsequent manipulation and analyses, including culture and growth, and analysis by biochemical and genomic techniques (Doležel et al 2007). This study demonstrates that flow cytometry may be used as a taxonomy tool, to distinguish among very close genotypes of a given species and, for the first time in plants, that this approach can also be put to profit for a characterization of the cytoplasmic acid phase and the concomitant production of secondary metabolites of interest in vitro, with or without elicitation. FCM also permits a detailed study of multiple optical parameters simultaneously, e.g., for the determination of the relative nuclear DNA content, genome size, and ploidy level for a large number of species in a short period of time (Zonneveld et al. 2005; Doležel et al. 2007; Ochatt 2008; Ochatt and Seguí-Simarro 2021).

FCM has shown that nuclear DNA content and ploidy level are closely related (DeLaat et al. 1987; Cvikrová et al. 2003; Loureiro et al. 2006; Doležel et al. 2007; Ochatt and Seguí-Simarro 2021). In addition, FCM has been used to analyze the composition of various tissues in terms of their chemical composition, including subcellular substances (Cvikrová et al. 2003; Loureiro et al. 2006) and cell wall fractions (Ochatt 2008), and also has applications in genome mapping (Jayakody et al. 2023). Anyway, the most frequent application of FCM remains the analysis of ploidy level (Ochatt and Seguí-Simarro 2021) and genome size (Kelly et al. 2012; Eicher et al. 2022) comparing the flow cytometry profile of the studied genotype with an internal standard of known ploidy and genome size.

The last update of the website of the Royal Botanic Gardens at Kew in London (accessible at http://www.kew.org/genomesize/homepage.html) indicates that the range of genome size variation in the plant kingdom extends to nearly 2000-fold (Leitch et al. 2019). Today, this constantly updated database compiles the DNA *C* values of nearly 2% of land plant species, i.e., about 9500 species. In this context, knowledge of the genome size of medicinal plant species is needed for genomic studies, aimed at performing full genome sequencing and also to study their transcriptome (Zhang et al. 2013; Mamgain et al. 2022).

Before this work, only five teams had undertaken the study of the relative DNA content and genome size in species of the genus *Salvia* (Maksimović et al. 2007; Munzbergova 2009; Siljak-Yakovlev et al. 2010; Temsch et al. 2010; Bou Dagher-Kharrat et al. 2013). Unfortunately, except for *S. glutinosa* (Siljak-Yakovlev et al. 2010) and for *S. brachyodon* and *S. officinalis* (Maksimović et al. 2007), in the remaining reports, species of the genus *Salvia* were simply listed as part of the flora of different regions and not described in much detail. This is to the best of our knowledge the first report for *S. hispanica* in this domain, and the results showed that the two genotypes studied were very close between each other in terms of genome size (0.4 pg 1C DNA for KruKam and 0.36 pg 1C DNA for Bio Planet), and smaller than data for other *Salvia* species published to date.

It has been convincingly shown that the nuclear DNA content of a given species is remarkably constant (Galbraith et al. 1983; Doležel et al. 2007; Ochatt 2008), while it may vary considerably among different taxonomically close species (Ochatt et al. 2013), and even among landraces and genotypes from a different ecotypic origin within a given species (Koné et al. 2007). In this context, *P. sativum* L. (pea) has become the most frequently used internal standard for flow cytometry studies due to the constancy in its genome size (Baranyi and Greilhuber 1995, 1996; Baranyi 1999). Nevertheless, even for pea, some differences were found in terms of 2C DNA content between reports (i.e., 8.37 pg DNA according to Marie and Brown (1993) but 9.05 pg DNA in Doležel et al. (1994)). This is why data for most species in the Kew Royal Botanic Gardens website (http://data.kew.org/cvalues/) as well as in Leitch et al. (2019) is usually given as the mean value and its range, as also adopted for *Salvia* in this study.

In these studies with chia, it was decided to assess the A-T/G-C ratio in addition to the ploidy level and genome size. This was undertaken by using the non-intercalating base-specific dyes DAPI (Kapuscinski 1995; Barow and Meister 2002; Doležel et al. 2007; Ochatt 2008) or CA3 (Vinogradov 1994) for the former and the intercalating and mostly base-independent dye PI (Greilhuber 2005) for the genome size.

Interestingly, studies on the AT/GC ratio in plants are sparse, even when different species are known to vary regarding the nucleotide base pair ratio of their genomic DNA (Marie and Brown 1993; Barow and Meister 2002), and this same approach has also been used in the past for the distinction between species and between related ecotypes of a given species (Koné et al. 2007; Ochatt et al. 2013). Also noteworthy, there is some controverse about the existence of a correlation between genome size and base pair ratio as claimed by Doležel et al. (2007) but not found by Barow and Meister (2002) who ascribed it to the non-random distribution of bases in the DNA molecule.

The rationale underlying the study of G-C in this study resides in the medicinal interest of chia, as guanine and cytosine are present in two distinct genomic chromosome regions, one mainly containing housekeeping and primary metabolism genes while the other is mostly responsible for the synthesis of secondary metabolites and hydrolytic enzymes (Bentley et al. 2002; Hopwood 2007). This has been studied mostly on *Streptomyces* (Medema et al. 2010), but the observations made can be safely extended to higher plants. The results of this study revealed that the *S. hispanica* genotypes have a G-C% slightly greater (< 1%) than observed for *S. brachyodon* and *S. officinalis* (Maksimović et al. 2007). A change range this small may be conducive to a loss of old amino acids and a gain of new ones and coupled with a reduced change in the amino acid composition and sequence (Du et al. 2018).

Although debated for a long time, it is accepted today that GC pairs are generally more stable than AT pairs, and GC-rich genomes are more adapted to high temperatures than AT-rich genomes (Hu et al. 2022). On the other hand, the influence of abiotic stress signals on secondary metabolites production in plants is well established for more than a decade (Akula and Ravishankar 2011). Moreover, it is beyond speculation that an increased G-C% in the genome will imply a larger production of secondary metabolites of interest, as observed with the in vitro tissues of KruKam and Bio Planet chia compared with tissues from in vivo grown plants of the same genotype. In vitro cultures obtained from the two genotypes of *S. hispanica* have a higher rosmarinic acid content compared with those found in plant material obtained from in vivo conditions (Motyka et al. 2023a, b). Rosmarinic acid content in non-elicited in vitro cultures for KruKam genotype (881.09 mg/100 g DW) was 4.5 times higher than that in tissues from in vivo grown plants of the same genotype (198.53 mg/100 g DW). For genotype Bio Planet obtained in vitro, the content of rosmarinic acid (838.14 mg/100 g DW) was almost 5 times higher compared with in vivo conditions (175.28 mg/100 g DW). In addition, elicited tissues of each genotype of *S. hispanica* contained a higher content of rosmarinic acid than non-elicited cultures, and this study proved that for both genotypes, CdCl_2_ at the lower concentration (500 μM) was better, whereas for YeE, the higher the concentration of elicitor used, the higher rosmarinic acid content.

This research has confirmed that the use of elicitors in the appropriate concentration can significantly contribute to increase rosmarinic acid production. The positive effect of elicitors on the production of secondary metabolites was also confirmed by other studies conducted on in vitro cultures of various species of *Salvia* (Attaran Dowom et al. 2022; Grzegorczyk-Karolak et al. 2022; Radomir et al. 2023; Rostami et al. 2022; Shoja et al. 2022; Szymczyk et al. 2021). The stimulation of rosmarinic acid production and accumulation by elicitors such as YeE has also been observed in the cell cultures of other plant species, including *Orthosiphon aristatus* (Mizukami et al. 1992), *Lithospermum erythrorhizon* (Sumaryono et al. 1991; Mizukami et al. 1993), *Coleus blumei* (Szabo et al. 1999), *Zingiber officinale* callus cultures (Ali et al. 2018), and *Polygonum multiflorum* beard root cultures (Ho et al. 2018). In a study conducted on hairy root cultures of *S. miltiorrhiza* to test the impact of adding YeE or silver ions (Ag^+^) on rosmarinic acid production, Yan et al. (2006) showed that the effect of elicitors on rosmarinic acid content depended on the elicitor type and dosage. Both elicitors increased rosmarinic acid accumulation as well as total phenolic content in hairy root cultures, but the synthesis was more significantly affected by YeE, in a concentration-dependent manner. The stimulating effect of YeE was stronger at larger doses, of 200 and 400 mg/L attaining 1.5- and 1.6-fold of the rosmarinic acid contents in the control after 4 and 8 days, respectively. Maximum amounts of rosmarinic acid were obtained in the presence of 200 mg/L YeE and at 15 mM Ag^+^. Moreover, the hairy root growth (root weight) was enhanced by the addition of YeE (200 mg/L) but not significantly affected by the addition of Ag^+^ (15 mM) (Yan et al. 2006). Sahu et al. (2013) also confirmed the positive eliciting effects of YeE, salicylic acid (SA), and methyl jasmonate (MeJa) on the rosmarinic acid production in shoot cultures of *Solenostemon scutellarioides* (*Lamiaceae*). Addition of MeJa (50 µM) and SA (50 µM) caused a 1.7- and 1.4-fold increase in rosmarinic acid accumulation within 24 h, respectively, while addition of YeE (100 µg/mL) showed a 1.5-fold increase in rosmarinic acid content after 72 h compared with non-elicited plants. YeE was found to be significantly effective between 50 and 100 µg/mL, and rosmarinic acid content remained nearly constant between days 1 and 3. Maximum rosmarinic acid concentration was achieved with 100 µg/mL YeE on day 3 (Sahu et al. 2013). Goncharuk et al. (2022) examined the elicitor effect of YeE in various concentrations (200–1000 mg/L) on the accumulation of phenolic compounds in flowering flax (*Linum grandiflorum* Desf.) cells cultured in vitro and their antiradical activity.

In this study, LyDR stain was used to determine the production by tissues of organic acids, including rosmarinic acid, with or without elicitation by YeE or CD. LyDR is a highly selective red stain comprising of a fluorophore linked to a weak base which is only partially protonated at a neutral pH and can hence freely pass across the cell membrane and label acid organelles in living cells at nanomolar concentrations. LyDR has been used several times in microscopic studies, e.g*.*, for the detection of autophagy-associated lysosomal activity in tissues of *Drosophila* such as the fat body, midgut, salivary gland, and ovary, but also in *Drosophila* cell cultures (DeVorkin and Gorski 2014). Importantly, studies using LyDR to date concerned animal cells only, and this study is, to the best of our knowledge, the first report on its use with higher plant cells and its analysis with flow cytometry. A reason could be that in studies with animal cells, LyDR was used to stain lysosomes, and there has been an intense controversy among cell biologists on whether plant cells do contain lysosomes or not until the convincing report by Swanson et al. (1998), showing that plant cell vacuoles can carry out lysosomal functions.

Against this background, chia tissues were stained with LyDR to measure the acid compartments that are formed after the stress induced by the addition of elicitors at a high concentration. An increased production of rosmarinic acid leads to a decrease of the cytoplasm pH and simultaneously generates a stress for the endoplasmic reticulum. Thus, the use of elicited material allows to see if there are differences in the production of secondary metabolites and also whether they are a response to stress (Al-Khayri et al. 2023). This increased production of rosmarinic acid would also entail an increased production of reactive oxygen species (ROS) which, in turn, imply an improved antioxidant activity to maintain redox homeostasis of the cytoplasm (Cruz de Carvalho 2008), cell membrane integrity (Traber and Stevens 2011), and, by extension, cell viability. These facts support the relevance of our studies on the endoplasmic reticulum stress and acid compartment formation within the context of evaluating the production of organic acids, in particular rosmarinic acid, following elicitation.

In conclusion, the studies aiming to unravel some of the most complex fundamental mechanisms underlying plant cytogenetics have been significantly aided by FCM. Today, the application of FCM to determine the ploidy level of tissues and plants after they have been manipulated in vitro or treated with mitogens has become commonplace. Moreover, FCM allows to reliably evaluate the ploidy level, nuclear DNA content, and division frequency (through detailed analysis of the cell cycle) for a comprehensive characterization of plants, tissues, and regenerants. This study shows that FCM may also be used as a taxonomy tool, to distinguish among very close genotypes of a given species and, for the first time in plants, that this approach can also be put to profit for a characterization of the cytoplasmic acid phase and the concomitant production of secondary metabolites of interest by LyDR-stained cells in vitro, with or without yeast extract (YeE) or cadmium chloride (CD) elicitation. Plant in vitro cultures of *S. hispanica* can be an innovative and useful source of secondary metabolites, as they contain almost 5 times more rosmarinic acid compared with plant material grown in vivo. The rosmarinic acid content differed between tissues of two *S. hispanica* varieties obtained in vivo and in vitro, treated with different elicitors at different concentrations. Elicited tissues of each genotype of *S. hispanica* had a higher content of rosmarinic acid compared with non-elicited cultures. This study demonstrates that for both genotypes, CD was better at the lower concentration (500 μM), while the reverse was observed for YeE where the higher concentration of elicitor used resulted in the higher rosmarinic acid content in the plant tissue tested.

## Supplementary Information

Below is the link to the electronic supplementary material.

Supplementary file1 (PDF 980 KB)

## Data Availability

Raw data were generated at the Department of Pharmaceutical Botany, Faculty of Pharmacy, Jagiellonian University Medical College, and Agroécologie, INRAE, Institut Agro, Univ. Bourgogne- Univ. Bourgogne Franche Comté. The data that support the findings of this study are available on request from the corresponding author (AS). The authors confirm that the data supporting the findings of this study are available within the article.
