# Supplementary material for: Distinction of chia varieties in vivo and in vitro based on the flow cytometry and rosmarinic acid production
Source: Appl Microbiol Biotechnol. 2024 May 20;108:337. doi: 10.1007/s00253-024-13171-w (PMC11106112; doi:10.1007/s00253-024-13171-w)
Supplement: Supplementary file 1 [file 253_2024_13171_MOESM1_ESM.pdf]

Applied Microbiology and Biotechnology

Supplementary material

Distinction of chia varieties in vivo and in vitro based on the flow cytometry and rosmarinic acid production

Sara Motyka[a,b], Agnieszka Szopa[a*], Sergio J. Ochatt[c]

[a] Chair and Department of Pharmaceutical Botany, Medical College, Jagiellonian University, Medyczna 9, 30-688 Kraków, Poland; *a.szopa@uj.edu.pl

[b]Doctoral School of Medical and Health Sciences, Medical College, Jagiellonian University, Łazarza 16, 31-530 Kraków, Poland; sara.motyka@doctoral.uj.edu.pl

[c]Agroécologie, INRAE, Institut Agro, Univ. Bourgogne, Univ. Bourgogne Franche-Comté, F-21000 Dijon, France; sergio.ochatt@inrae.fr

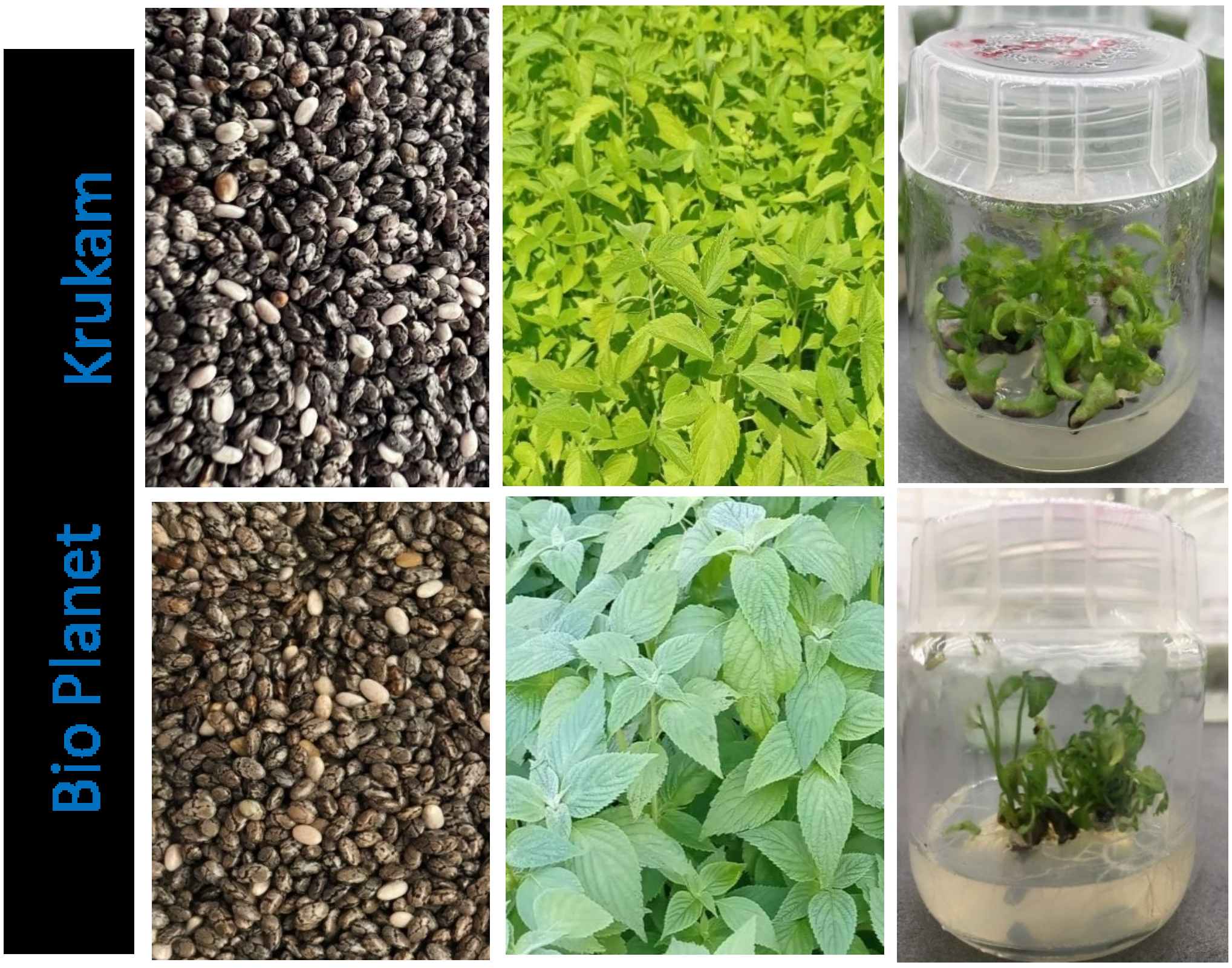

Figure S1. The morphological appearance of seeds, leaves, and microshoot cultures obtained from the two *S. hispanica* varieties.

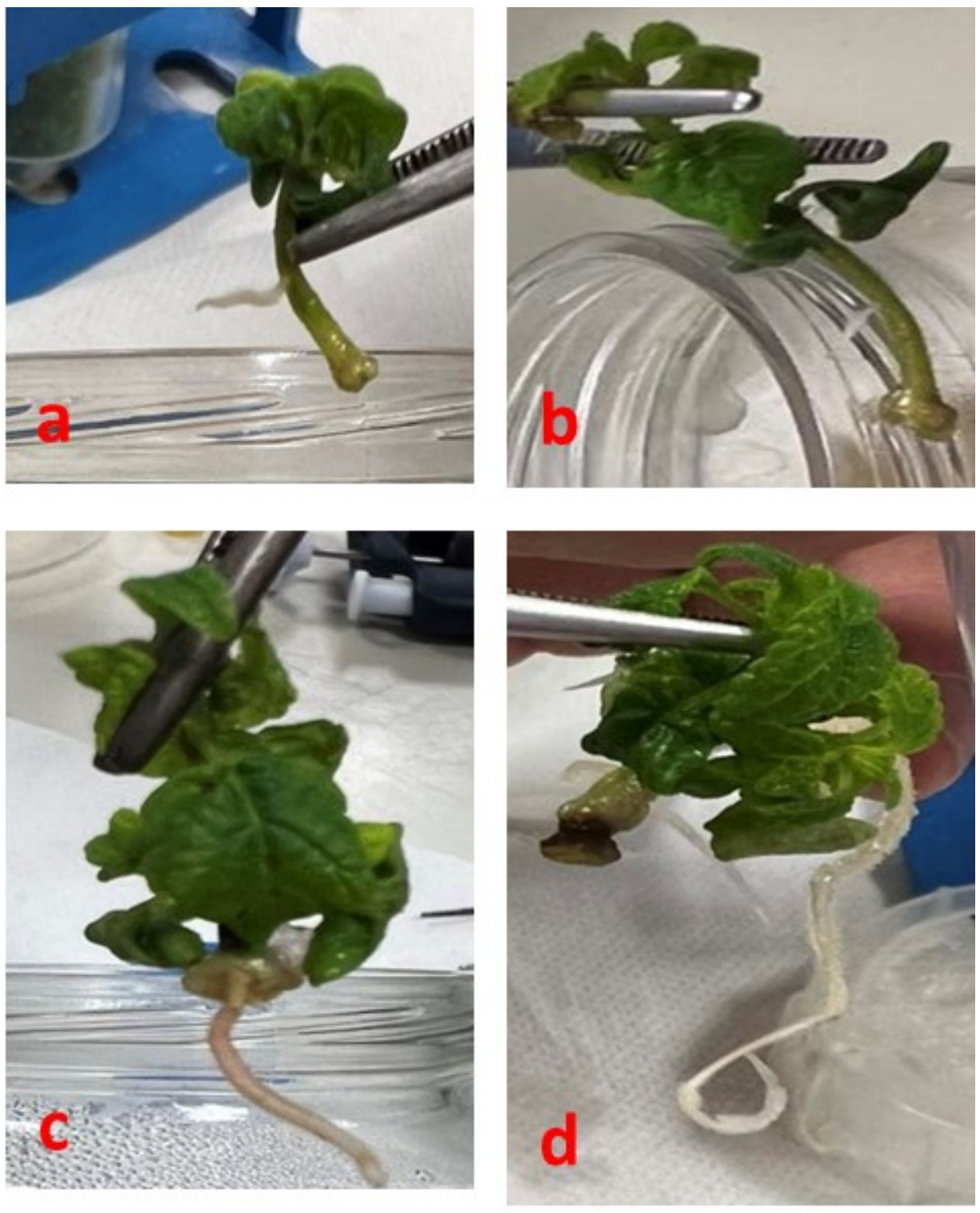

Figure S2. Differences in the appearance of roots of in vitro cultures of *S. hispanica* elicited with YeE at 500 mg/ (a, Krukam; c, Bio Planet) and 1000 mg/l (b, Krukam; d, Bio Planet).
